# Personal protection with PBO-pyrethroid synergist-treated nets after 2 years of household use against pyrethroid-resistant *Anopheles* in Tanzania

**DOI:** 10.1186/s13071-021-04641-5

**Published:** 2021-03-10

**Authors:** Jackline L. Martin, Franklin W. Mosha, Eliud Lukole, Mark Rowland, Jim Todd, Jacques D. Charlwood, Jacklin F. Mosha, Natacha Protopopoff

**Affiliations:** 1 Kilimanjaro Christian Medical University College, Moshi, United Republic of Tanzania; 2 National Institute for Medical Research-Mwanza Centre, Mwanza, United Republic of Tanzania; 3 London School of Hygiene and Tropical, London, United Kingdom

**Keywords:** Personal protection, *An. gambiae*, *An. funestus*, Insecticide resistance, Olyset plus, Olyset net, PBO, Piperonyl butoxide, Pyrethroid, Tanzania

## Abstract

**Background:**

The spread of pyrethroid resistance in malaria vectors threatens the effectiveness of standard long-lasting insecticidal nets (LLIN). Synergist nets combine pyrethroid (Py) and piperonyl-butoxide (PBO) to enhance potency against resistance mediated by mono-oxygenase mechanisms. Our project assessed personal protection of the World Health Organization first-in-class PBO-Py LLIN (Olyset Plus) versus the standard LLIN (Olyset net) against pyrethroid-resistant *Anopheles gambiae* sensu lato (s.l.) and *An. funestus* in North-West Tanzania after 20 months of household use.

**Methods:**

From a household survey, 39 standard Olyset net and 39 Olyset Plus houses were selected. The physical integrity and hole index (HI) of the nets were assessed, and resting mosquitoes were collected from inside nets and from room walls. The indoor abundance was estimated using CDC light traps and species identified using PCR. The bioefficacy of PBO and standard LLINs against wild *Anopheles* was assessed using 30-minute cylinder bioassays.

**Results:**

Of 2397 *Anopheles* collected, 8.9% (*n* = 213) were resting inside standard Olyset nets, while none were found inside Olyset Plus nets (PBO-Py LLINs) of any HI category. Resting density of blood-fed mosquitoes was higher on walls of sleeping rooms with Olyset nets compared to Olyset Plus (0.62 vs 0.10, density ratio [DR]: 0.03, 95% CI 0.01–0.13, *p* < 0.001). Mosquitoes were found inside Olyset nets of all WHO HI categories, but more were collected inside the more damaged nets (HI ≥ 643) than in less damaged (HI 0–64) nets (DR: 6.4, 95% CI 1.1–36.0, *p* = 0.037). In bioassay, mortality of *An. gambiae* s.l. was higher with Olyset Plus than with Olyset nets for new nets (76.8% vs 27.5%) and nets used for 20 months (56.8% vs 12.8%); similar trends were observed with *An. funestus*.

**Conclusion:**

The PBO-Py LLINs provided improved protection after 20 months of household use, as demonstrated by the higher bioassay mortality and absence of pyrethroid-resistant *An. gambiae* sensu stricto (s.s.) and *An. funestus* collected from inside Olyset Plus nets, irrespective of HI category, as compared to Olyset nets.
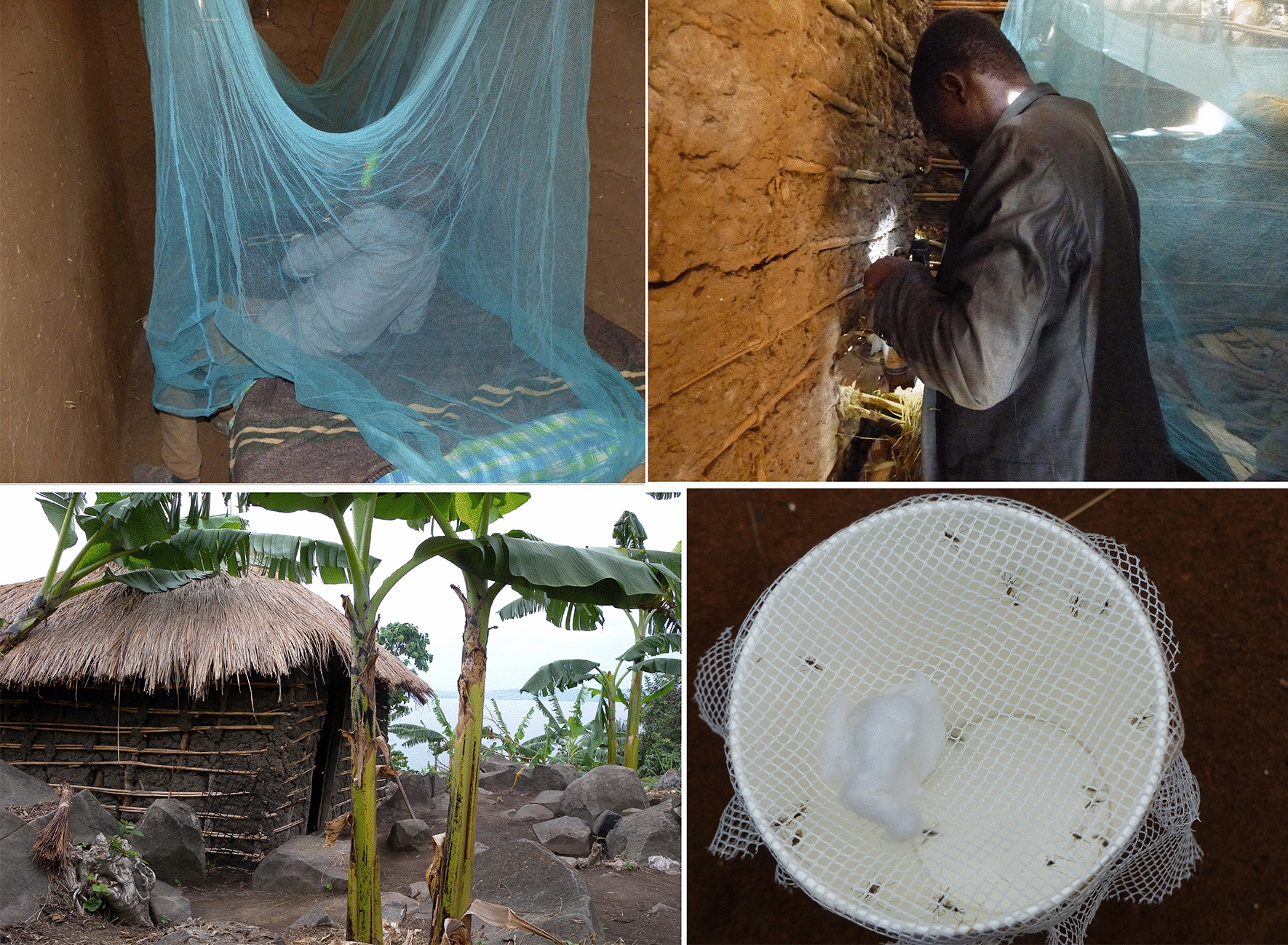

## Background

Long-lasting insecticidal nets (LLINs) are the cornerstone of malaria control in sub-Saharan Africa. The global malaria burden was reduced by 40% between 2000 and 2015, with insecticide-treated nets (ITNs) and LLINs making the largest contribution to control [1]. Since 2015, the annual malaria burden has not fallen any further in Africa [2]. The reasons for this are complex. While LLINs may provide personal protection for users even after becoming holed due to their insecticidal and excito-repellency effects [3], it has been shown that highly resistant phenotypes can penetrate the LLIN holes more readily to blood-feed and be found resting on inner surfaces [4, 5]. In such conditions, protective efficacy from malaria may be reduced when the physical condition of the net deteriorates [6]. Following the selection of high-level pyrethroid resistance arising from a combination of L1014S knock-down resistance (*kdr*) mutation and mono-oxygenase metabolic mechanisms, also known as cytochrome P450s, in North-West Tanzania, even new intervention with standard LLINs may not reduce malaria transmission substantially [7–9].

In response to the rapid spread of pyrethroid resistance across Africa, the World Health Organization (WHO) has encouraged manufacturers to develop new types of LLINs that contain active ingredients with new modes of action to address the problem. Olyset Plus is a new-generation LLIN which incorporates pyrethroid permethrin and synergist piperonyl butoxide (PBO) to counter resistance to pyrethroids in mosquitoes caused by cytochrome P450-based metabolic mechanisms [10]. PBO inhibits the P450 enzymes which are responsible for detoxification of the pyrethroid before the neurotoxin reacts with its target site. In a cluster randomised trial (CRT) conducted in the same area as the present study, full coverage with PBO-Py LLINs (Olyset Plus) provided community protection against malaria for up to 2 years of use [11]. A further study, this one in Uganda, showed higher efficacy of PBO-Py LLINs compared to standard LLINs over the 18 months of the trial [12].

The aim of this study was to examine in an area of high pyrethroid resistance in North-West Tanzania the relationship between net holes and blood feeding of mosquitoes collected from inside pyrethroid-only and PBO-Py LLINs and from room surfaces containing these nets. The study provides evidence for differences in mosquito feeding success between Olyset Plus and standard Olyset nets under household conditions that serves as a proxy for personal protection from Olyset Plus after 20 months of use.

## Methods

### Study area

This study was embedded within a CRT whose main aim was to assess the community effect on malaria of a PBO-Py LLIN (Olyset Plus), in comparison with a standard pyrethroid LLIN (Olyset net), over 3 years of use [11]. The present study was conducted 20 months after the distribution of Olyset and Olyset Plus nets between October 2016 and February 2017. The data collection was from four villages in Muleba district; Kyamyorwa and Ntungamo received Olyset Plus nets, and Kakoma and Kabirizi received Olyset nets. The four villages were selected because they had the highest density during the second post-intervention trapping year. Details of the study area have been reported previously [13]. In brief, the main malaria vectors in Muleba were *An. gambiae* s.s. (92%), *An. funestus* s.l. (4%) and *An. arabiensis* (4%) [11]. Pyrethroid resistance frequency was over 90% in *An. gambiae* s.l. and 45% in *An. funestus* s.l. exposed to the WHO diagnostic dose of permethrin (0.75%). Addition of PBO in synergist bioassay resulted in restoration of susceptibility in *An. gambiae* which indicates the involvement of cytochrome P450 in pyrethroid resistance [9].

### Household and net survey

Ten to 12 houses were randomly selected from each of the four villages, and a maximum of two study nets in use were selected from each house at random until 40 nets of each type were reached. Information was collected from each house on household characteristics, LLIN ownership and usage. The selected LLINs were fitted over an Ifakara-style frame [14] and examined for presence, number, position and size of holes in accordance with the WHO LLIN guidelines [15]. Holes were categorised into four size classes (hole size 1 = 0.5–2 cm diameter; 2 = 2–10 cm; 3 = 10–25 cm and 4 = larger than 25 cm) and weighted to estimate the hole area and hole index (HI) of each net [15]. HI was then allocated into three categories: “good” condition (HI 0–64), “acceptable” (65–642) or “torn” (≥ 643).

### Mosquito collection

Repeated mosquito collections were undertaken in each house during 6–10 visits over 5 months to obtain sufficient data for statistical analysis. Resting mosquito collection was conducted for 6 days in the villages with standard LLINs and up to 10 days in villages with PBO-Py LLINs due to lower number of mosquitoes collected per day. Mosquitoes were collected from inside the selected study nets, and walls of rooms where the nets were installed were searched for mosquitoes using mouth and prokopack aspirators. An additional two nights of collection were done after completion of the resting collection visits using Centers for Disease Control and Prevention (CDC) light traps installed at the foot of a bed fitted with the selected study net and run from 7 pm to 7 am on each night. This was to gain further insight on overall mosquito densities and species entering the selected houses.

All mosquitoes collected were identified to species [16] and categorised to gonotrophic status microscopically (i.e. unfed, freshly fed, semi-gravid and gravid). *An. gambiae* s.l. were further identified to species using a real-time PCR Taq Man assay to distinguish *An. gambiae* s.s. from *An. Arabiensis* [17].

### Bioefficacy of the LLINs after 0 and 20 months

The residual bioefficacy of the Olyset and Olyset Plus nets collected after 20 months of use was investigated on wild-caught *An. funestus* s.l. and *An. gambiae* s.l. using WHO cylinder test kits lined with the netting sampled from the study nets [18]. Netting was stapled to backing paper the same size as WHO test paper before insertion into the cylinder. Wild adult *Anopheles* of indeterminate age were collected using a prokopack and manual aspirator from inside houses (collection houses were different from the study houses selected for CDC light trapping and indoor resting collections) and were provided with glucose for 2–3 days to allow for blood digestion before testing. *Anopheles* were then exposed for 30 minutes to the net samples in the WHO cylinders, and mortality was recorded 24 hours later. Four to five replicates of 20 mosquitoes were exposed to each treatment. Bioefficacy tests were also carried out on unused, unwashed Olyset Plus and Olyset nets.

### Data analysis

All data analysis was done using Stata version 13. Household characteristics, building materials, presence/absence of eaves, mosquito abundance, hole index and hole area were summarised according to net type and age.

Negative binomial regression was used to estimate the association between hole index and density of blood-fed *Anopheles* mosquitoes, adjusting for date and clustering by mosquitoes per net and repeated measures using robust standard errors.

## Results

### Household and net characteristics

The net surveys were carried out in 20 households with standard Olyset nets and 23 households with Olyset Plus nets. A total of 39 nets of each type were examined. Household and net characteristics were similar in the two groups. Average family size was 6.3 (standard deviation 1.8) in Olyset net households and 7 (standard deviation 2.6) in Olyset Plus households. In Olyset net villages, 15 (75.0 %, 95% CI 51.1–89.6) of selected houses had mud walls and 11 (55% 95% CI 32.8–75.4) had open eaves, while in Olyset Plus villages, 22 (96.7%, 95% CI 73.2–99.4) had mud walls and 13 (56.5%, 95% CI 34.8–76.0) had open eaves. Over 80% of each net type had been in use for more than 20 months, and the median hole index and hole area at 20 months were similar between net types (Table 1).

**Table 1:** Insecticide-treated net characteristics and age in months when sampled

	Olyset net		Olyset Plus	
	0–5 months	20–21 months	0–5 months	20–21 months
Nets with at least one hole %, (*n*/*N*)	33% (2/6)	100% (33/33)	0% (0/4)	94% (33/35)
Hole area (cm^2^)				
Mean (std. dev.)	190 (417)	874 (1237)	–	1526 (2689)
Median (IQR)	–	573 (65–1104)	–	640 (95–1562)
Hole index				
Mean (std. dev.)	155 (340)	712 (1008)	–	1243 (2191)
Median (IQR)	–	467 (53–898)	–	521 (77–1273)
Hole index category % (*n*)				
0–64	67% (4)	30% (10)	100% (4)	20% (7)
65–642	17% (1)	33% (11)	0% (0)	31% (11)
≥ 643	17% (1)	36% (12)	0% (0)	49% (17)

### Mosquito densities according to net type and collection method

At 20 months post-intervention, when the study was carried out, a total of 2397 Anopheline mosquitoes were collected from standard Olyset nets and Olyset Plus nets and rooms using aspirators and light traps (Table 2). In CDC light traps, a higher density of mosquitoes were collected from households with standard Olyset nets (mean:17.5) than with Olyset Plus (mean: 6.4, DR: 0.37, 95% CI 0.16–0.83, *p* value = 0.015).

**Table 2:** Indoor density of *Anopheles* mosquitoes according to collection method and net type

	Olyset net			Olyset Plus		
	Light trap	On wall	Inside net	Light trap	On wall	Inside net
Total number of collection events	40	240	239	41	300	300
Mean no. Anophelines per collection (*N*)	17.5 (699)	3.0 (719)	0.9 (213)	6.4 (264)	1.7 (502)	0
Proportion *An. gambiae* s.l. (*n*)	56.7 (396)	54.7 (393)	61.5 (131)	86.7 (255)	98.7 (465)	0
Proportion *An. funestus* (*n*)	43.3 (303)	45.3 (326)	38.5 (82)	13.3 (39)	1.3 (6)	0
Total *An. gambiae* s.l. tested for species		175	138		126	0
Proportion *An. arabiensis* (*n*)		1.1% (2)	0.7% (1)		41.3% (52)	0

*N* = total *Anopheles* collected

For standard Olyset nets, a mean of 0.9 mosquitoes were collected per net and three mosquitoes per room from wall collection. Overall, 13.1% (*n* = 213) were collected from inside standard Olyset nets, 42.9% (*n* = 699) from CDC light traps and 44.1% (*n* = 719) from the room walls (Table 2). Among the 213 mosquitoes collected from inside the Olyset nets, 91% (*n* = 193) were freshly blood-fed.

Zero mosquitoes were collected from inside Olyset Plus nets (Table 2). Mean number of blood-fed *Anopheles* resting on the walls was also much lower in rooms with Olyset Plus (mean: 0.10) compared to Olyset nets (mean: 0.63, DR: 0.16, 95% CI 0.04–0.69, *p* value 0.015). The two parameters are both indicative of high levels of personal protection.

The overall proportion of *An. funestus* collected relative to *An. gambiae* s.l. was much less in villages and houses with Olyset Plus (5.9%, 45/766) than in villages and houses with standard Olyset net (43.6%, 711/1631). Among the *An. gambiae* s.l. resting on the wall identified to species, a much higher proportion of *An. arabiensis* (41.3%) relative to *An. gambiae* s.s. were found in households with Olyset Plus than in households with standard Olyset nets (1.1%).

### Blood-fed *Anopheles* mosquitoes collected while resting inside nets and on walls

The average number of *Anopheles* mosquitoes found inside 20-month-old Olyset nets was higher in torn LLINs with HI ≥ 643 than inside standard Olyset nets in “good” condition (HI 0–64: DR: 6.38, 95% CI 1.11–36.0, *p* value: 0.037) after adjusting for collection date (Table 3). No significant difference in density was found between nets in good and acceptable conditions. Most notably, no mosquitoes were found resting in Olyset Plus nets irrespective of net condition (Table 2). Density of blood-fed mosquitoes resting on room walls increased as hole index increased in rooms containing an Olyset net, while resting density remained stable across the three HI categories in Olyset Plus rooms (Table 3).

**Table 3:** Mean density of blood-fed *Anopheles* found inside Olyset net and resting on the walls of Olyset net and Olyset Plus rooms (≥ 20 months) by hole categories

Hole index category	Total collection	Mean *Anopheles* inside net	DR^a^	95% CI	*p* value	Mean *Anopheles* resting on wall	DR^a^	95% CI	*p* value
Olyset net									
0–64	60	0.5	1			0.05	1		
65–642	66	0.2	0.43	0.06–3.30	0.421	0.23	4.39	0.51–37.8	0.178
≥ 643	72	2.0	6.38	1.11–36.0	0.037	1.68	39.18	6.33–242.5	<0.001
Olyset Plus									
0–64	54	0				0.09	1		
65–642	90	0				0.10	1.14	0.37–3.47	0.819
≥ 643	126	0				0.12	1.97	0.56–6.91	0.289

^a^ Density Ratio (DR) adjusted for collection date

### PBO-pyrethroid LLIN bioefficacy monitoring

Cylinder bioassays with wild, field-caught mosquitoes exposed to new Olyset nets showed mortality of 27.5% in *An. gambiae* s.l. and 27.2% in *An. funestus* (Table 4). After 20 months of use, the bioassay mortality decreased to 12.8% in *An. gambiae* s.l. and to 2.3% in *An. funestus.* The percentage mortality on exposure to new unwashed Olyset Plus nets was 76.8% in *An. gambiae* s.l. and 81.1% in *An. funestus*, approximately three times greater compared to Olyset nets. After 20 months of use, the mortality on Olyset Plus decreased to 56.8% in *An. gambiae* s.l. and 25.3% in *An. funestus*, but mortality was still proportionately higher on Olyset Plus than on Olyset nets of similar age.

**Table 4:** Mortality of wild *An. gambiae* s.l. and *An. funestus* after 30-minute exposure and 24 hours holding with a new and a 20-month-old Olyset net and Olyset Plus net in WHO cylinder bioassays

	Olyset net		Olyset Plus	
	0 months	20 months	0 months	20 months
*An. gambiae*				
Total tested	80	78	82	81
Percent mortality at 24 hours (%)	27.5	12.8	76.8	56.8
95% CI	17.3–37.7	10.4–15.3	67.6–86.1	46.9–66.6
*An. funestus*				
Total tested	92	88	95	91
Percent mortality at 24 hours (%)	27.2	2.3	81.1	25.3
95% CI	22.5–31.8	0–5.1	65.2–96.9	8.7–41.9

Zero mortality was observed after exposure to untreated nets in *An. gambiae* (tested = 79) and *An. funestus* (tested = 91)

## Discussion

The study showed that after 20 months of field use, the PBO-Py LLIN (Olyset Plus) continued to provide improved personal protection compared to the standard pyrethroid LLIN (Olyset net). There were no mosquitoes to be found resting inside Olyset Plus nets irrespective of LLIN condition or hole index, while inside Olyset nets, blood-fed *Anopheles* mosquitoes were found resting in nets of all physical conditions, increasing to a mean of two *Anopheles* per net in those of the highest hole index category.

According to WHO, a hole index that exceeds category HI 643 is considered unserviceable or non-protective in a standard pyrethroid LLIN [19]. The presence of blood-fed mosquitoes in a standard pyrethroid LLIN with a hole index less than this upper limit (i.e. across the range of HI 0–642 rather than just ≥ 643) indicates that the categorisation of hole index adopted by WHO to distinguish between serviceable and non-serviceable categories has lost validity with respect to standard LLINs in environments that include a high proportion of highly resistant vectors. Similarly, in an area of high pyrethroid resistance in neighbouring Kenya, mosquitoes were collected inside standard LLINs in good and acceptable conditions, while none were found inside standard nets in an adjacent area comprised of susceptible mosquitoes [5].

The findings of the present study are also consistent with the observations made in the CRT, conducted in the same locality, in which no decrease in malaria prevalence occurred in the standard LLIN arm over the first 2 years of the trial, whereas in the PBO-Py LLIN arm, malaria prevalence decreased by almost one-half during the first 2 years [11]. The complete absence of mosquitoes in the Olyset Plus nets of any hole index category in the present household study after 20 months and low frequency of blood-fed mosquitoes on walls may be interpreted as restoration of protection by the PBO-Py LLIN despite the wide range of hole indices to be seen in these nets after almost 2 years of use. Pyrethroid and PBO content were not assessed for these nets; however, other study nets collected at the same time showed pyrethroid content of 16.7 g/kg in Olyset and 12.2 g/kg in Olyset Plus nets, while PBO content was 1.6 g/kg compared to 9.2 g/kg in new Olyset Plus nets [11], suggesting that PBO could still provide enhanced protection even at this much-reduced concentration. This restoration of protection against resistant mosquitoes seems analogous to the historic protection that was achieved when pyrethroid treatment of mosquito nets was first introduced 30 years ago to render untreated mosquito nets more protective against the pyrethroid-susceptible vector populations that were prevalent then [3]. With respect to PBO-Py LLINs, which are now being rolled out in preference to standard LLINs in many countries with pyrethroid-resistant vectors, the HI categorisation and absence of mosquitoes collected inside nets may still serve as a useful predictor of protection elsewhere in Africa until such time that resistance evolves against this class of net. When that point is reached, such highly resistant mosquitoes are predicted to survive and to be found resting inside PBO-Py LLINs.

CDC light trap surveillance was deployed to record the density of house-entering mosquitoes in Olyset net and Olyset Plus houses 20 months post-intervention. It was not our aim to have the study villages balanced in terms of mosquito density pre-intervention. Pre-intervention, the baseline density of *Anopheles* collected in CDC light traps in the two villages receiving Olyset Plus was 107.3 (median: 72) per night and 17.6 (median: 2) in the two Olyset net villages, which is an even greater difference than that seen 20 months after (Olyset Plus nets, mean 6.4; Olyset nets, mean 17.5) due to the cumulative effect of Olyset Plus on mosquito density post-intervention [11]. This re-emphasises the two effects of Olyset Plus compared to Olyset nets: greater *An gambiae* population control at the community level and enhanced personal protection for PBO-Py LLIN users.

Differences in blood feeding success between Olyset and Olyset Plus nets and, by inference, differences in personal protection have also been observed in experimental hut trials in pyrethroid-resistant areas elsewhere [10, 20]. While no contemporary experimental hut trial of Olyset Plus nets versus standard Olyset nets has yet been reported in Tanzania against a resistant population of *Anopheles gambiae*, experimental hut trials conducted in the pyrethroid-resistant West African country of Burkina Faso showed a significantly reduced blood feeding rate in unwashed Olyset Plus nets compared to Olyset nets [20]. In Benin, blood feeding rate was higher in huts with Olyset nets washed 20 times, which is equivalent to at least 2 years and probably 3 years of use, compared to Olyset Plus nets according to WHO’s proxy estimation of net longevity [10]. In the same experimental hut trial, it was also shown that the mortality of free-flying resistant mosquitoes induced by Olyset Plus (81%) was approximately double that of Olyset nets (42%), and this ratio did not change significantly after standardised washing over 20 cycles [10]. In a meta-analysis of experimental hut studies of several brands of PBO-Py LLINs, this new class of net was shown to induce 0.60 times less blood feeding than standard pyrethroid-only LLINs and to kill 1.85 times more pyrethroid-resistant mosquitoes [21]. In the context of the present Tanzanian study, major differences in mortality in residual bioassay were observed between Olyset Plus and standard Olyset nets (~ 50% difference for both *An. gambiae* s.l. and *An. funestus*) after 20 months of use. Of course, differences in mortality are more an indicator of toxicity and reduced mosquito longevity than of reduced blood feeding rate or personal protection. Nevertheless, the consistency in the trends in West African hut trials and Tanzanian household trials points towards higher efficacy of the PBO-Py LLINs compared to standard LLIN after 2 years of use.

The protection arising from the interaction between PBO and pyrethroid could be due to inhibition of metabolic resistance by PBO, leading to restoration of enhanced knock-down and mortality. However, the excito-repellency of mosquitoes away from the net after contact with the PBO-pyrethroid surface may be more pertinent to personal protection than the classical synergism of pyrethroid resistance. For instance, LeClair et al. [13] observed that mosquitoes entering bedrooms were not killed after contact with Olyset Plus but showed increased excitability and a heightened escape reaction and capture rate in light traps present in the same room. More studies would be necessary to unravel the effects of PBO-Py LLINs on mosquito behaviour and synergism of metabolic resistance mechanisms.

The anthropophilic *An. gambiae* s.s. was the predominant vector species. A high proportion of *An. arabiensis* was collected from rooms with Olyset Plus nets compared to rooms with Olyset nets. An earlier CRT in this area reported limited involvement of *An. arabiensis* in malaria transmission based on its lower sporozoite rate and entomological inoculation rate (EIR) [22]. Over a decade ago, in response to the successful control of the primary vector *An. gambiae* s.s. in South-eastern Tanzania following increased coverage of standard LLINs in the universal coverage campaign [23], the ratio of *An. arabiensis* to *An. gambiae* s.s. shifted towards higher *An. arabiensis*. That species shift has been attributed in part to the more zoophilic *An. arabiensis* being less effectively controlled by standard LLINs than *An. gambiae* s.s. in sympatric populations of the species complex [24]. In the present study, the control of *An. funestus* and, to a lesser extent, of *An. gambiae* s.s. at both community and household levels by PBO-Py LLINs (and the failure to control these species in villages and households of the standard LLIN arm) may indicate the beginning of a species shift from *An. funestus* and *An. gambiae* s.s. to *An. arabiensis* in Olyset Plus net villages in NW Tanzania. However, no firm conclusion can be drawn due to the lack of data about species composition resting on the walls in these four villages at baseline.

For the comparison of residual activity (bioassay mortality) of Olyset Plus and Olyset nets, a WHO cylinder test with a 30-minute exposure was used [18]. The Olyset net performs poorly in the cone test even against susceptible mosquitoes (due to high excito-repellency of permethrin) with 3-minute exposure. In the more recent WHO 2013 guideline for evaluating LLINs [15], the tunnel test is proposed over the cone if cone mortality is low with a 3-minute exposure using susceptible mosquitoes. It is however difficult to test wild mosquitoes in tunnels and get them to host-seek or feed on guinea pigs. Cylinder tests were therefore preferred as bioassays on resistant mosquitoes but using a longer exposure than 3 minutes. Thirty minutes of exposure was used, as this is standard practice for indoor residual spraying (IRS) bioassays, and there is evidence for longer natural exposure time on nets in experiment huts when mosquitoes are resistant [25]. In the present study, even with 30-minute exposure, the cylinder test managed to achieve only 27.5% mortality against resistant *An. gambiae* with a new Olyset and only 12.8% mortality on a 20-month-old Olyset net. Mortality rates of *An. gambiae* s.l. and *An. funestus* in Olyset Plus nets declined in cylinder bioassays after 20 months of use. Despite this, the PBO-Py LLINs were still protective against mosquito feeding under field conditions.

## Conclusion

The PBO-Py LLINs (Olyset Plus) provided improved protection after 20 months of household use, irrespective of hole index, as demonstrated by higher bioassay mortality and the absence of pyrethroid-resistant *An. gambiae* s.s. and *An. funestus* collected from inside Olyset Plus nets as compared to collections from inside standard LLINs after this period of use.

## Data Availability

The data sets generated and/or analysed during the current study are not public but are available from the corresponding author on reasonable request.
